# Tracking biochemical indicators of air pollution exposure in acute cardiorespiratory emergencies: a scoping review

**DOI:** 10.3389/fpubh.2026.1892038

**Published:** 2026-09-10

**Authors:** Davide Campagna, Caterina Ledda, Angela Trovato Salinaro, Francesca Cucuzza, Giuseppe Carpinteri, Massimo Caruso, Cinzia Lombardo, Elisa Trovato, Gabriella Lupo

**Affiliations:** 1 Department of Clinical and Experimental Medicine, School of Medicine, University of Catania, Catania, Italy; 2 Emergency Department-“Policlinico-San Marco” Teaching Hospital of Catania, Catania, Italy; 3 Department of Biomedical and Biotechnological Sciences, School of Medicine, University of Catania, Catania, Italy; 4 Internal Medicine Unit-“Policlinico-San Marco” Teaching Hospital of Catania, Catania, Italy

**Keywords:** air pollution, biochemical markers, cardiovascular emergencies, emergency department, inflammation, respiratory diseases, short-term exposure

## Abstract

**Background:**

Short-term ambient air pollution is a known trigger for acute cardiovascular and respiratory events. Biochemical markers help reveal pollution-induced physiological changes during emergency presentations, though evidence remains fragmented.

**Objectives:**

This scoping review mapped existing literature on short-term biochemical responses to air pollution in patients presenting acutely with cardiorespiratory conditions.

**Eligibility criteria:**

Eligible studies were human studies assessing at least one biochemical marker in people presenting to emergency departments after short-term exposure to outdoor air pollution.

**Sources of evidence:**

Five databases (PubMed, Scopus, Web of Science, Embase, CINAHL) were searched from 2000 to 2025.

**Charting methods:**

Data were extracted using the PCC framework and summarized in structured tables.

**Results:**

Twelve studies met criteria. PM_2.5_, PM10, and NO_2_ were most frequently linked to inflammatory and coagulative markers such as CRP, IL-6, D-dimer, and troponins, often with effects seen within hours to days.

**Conclusions:**

The mapped evidence suggests that short-term air pollution exposure is associated with acute biochemical alterations. Standardized exposure windows, harmonized biomarker panels, and more detailed subgroup analyses are needed before these markers can support emergency-care pathways and environmental health policy.

## 1 Introduction

Research consistently identifies PM_2.5_, NO_2_, and O_3_ as the air pollutants most commonly associated with acute cardiorespiratory presentations. Among these, PM_2.5_ shows the strongest correlation with cardiovascular hospital admissions across multiple studies (1) and is also linked to increase in-hospital mortality (2). NO_2_ demonstrates robust associations with cardiac conditions, including myocardial infarction and emergency department visits (2–4). In contrast, O_3_ is most consistently linked to respiratory outcomes, though it is also significantly associated with acute myocardial infarction (3, 5). These pollutants have been shown to influence coagulation pathways and respiratory function across a range of acute clinical presentations (6). Biochemical alterations commonly observed include increased inflammatory markers; for instance, particle number concentrations are linked to airway inflammation, while organic carbon, sulfate, and nitrate correlate with cardiovascular biomarkers such as C-reactive protein (CRP) and fibrinogen (7).

In cardiovascular research, a broad range of biochemical markers have been studied, especially those related to inflammation, oxidative stress, and myocardial injury. Cardiac troponins remain the diagnostic gold standard for myocardial infarction, albeit with certain limitations (Ralapanawa and R, 2022). Inflammatory markers such as CRP, IL-6, TNFα, and fibrinogen have been widely assessed (8, 9), while oxidative stress indicators include malondialdehyde (MDA), lipid peroxides, and oxidized proteins (10). Enzymatic markers such as CK-MB, AST, and LDH are also used to detect myocardial injury (11). Additional biomarkers like BNP, myeloperoxidase, and soluble CD40 ligand further enhance our understanding of cardiovascular pathophysiology (12, 13).

The temporal dynamics between air pollution exposure and biochemical alterations in emergency presentations are well-documented. Studies report increased cardiovascular and cerebrovascular emergency visits within 0–6 h following particulate exposure, with odds ratios of 1.04 for both conditions (14). A 10 μg/m^3^ increase in particulate matter correlates with a 1.97% rise in circulatory emergency calls the same day, and a 1.95% increase in respiratory-related calls within 48 h (15). In pediatric asthma cases, the strongest correlations with air pollution occur on the day of emergency presentation (16). Mechanistic studies suggest that pulmonary inflammation and oxidative stress are activated within 24 h, while coagulation pathways evolve over 2–3 days (17). Endocrine effects can emerge even faster, within 24 h or less (18). Biomarkers offer a temporally integrated assessment of bioavailable contaminant levels (19).

Despite shared features, asthma and COPD trigger distinct biochemical and inflammatory responses. Metabolomic profiling can differentiate between these conditions and healthy controls, with markers such as hypoxanthine elevated in asthma and purine metabolism alterations being characteristic (20). Urinary metabolomics distinguishes asthma from COPD with over 90% accuracy (21). Their inflammatory profiles also diverge: asthma is typically associated with eosinophils, mast cells, and Th2 lymphocytes, whereas neutrophils and macrophages pre-dominate in COPD (22, 23).

Certain populations show heightened vulnerability to air pollution in emergency contexts. Older adult individuals, infants, and those with pre-existing cardiopulmonary diseases experience greater morbidity and mortality from particulate exposure (24). People with diabetes show a twofold increase in heart disease admissions due to PM_10_ exposure compared to people without diabetes (25). Others at increased risk include individuals with respiratory infections, asthma, heart failure, and cardiac conduction disorders (26). The cardiovascular impact of PM_2.5_ is also more pronounced in women, racial/ethnic minorities, and those with low socioeconomic status (27). Interestingly, younger people with heart failure with reduced ejection fraction and no prior admissions are especially sensitive to PM_2.5_ exposure (28). Additional vulnerable groups include pregnant women and individuals with pulmonary diseases (29, 30).

Studies on exposure assessment and biomarker evaluation utilize a wide range of methodologies. Proximity models, air dispersion models, and hybrid satellite-based approaches are commonly employed, with the latter offering improved spatial resolution (31, 32). Recent advances include GPS tracking, low-cost indoor sensors, and interpolation models, particularly in pregnancy-related studies (33). For biochemical endpoints, over 234 biomarkers have been identified: 70 measure exposure (mostly related to PAHs), while 164 assess oxidative stress, inflammation, and DNA damage (34). Untargeted metabolomics further elucidates pollutant-induced metabolic perturbations (35). Non-invasive markers and human biomonitoring tools are increasingly used in field studies (31, 36).

Biochemical markers are also gaining importance as early diagnostic and prognostic tools in emergency medicine. Cardiac troponins and CK-MB remain central to myocardial infarction diagnosis, while newer markers like H-FABP, BNP, and Pro-BNP support rapid cardiovascular risk assessment.

Despite this growing body of research, significant knowledge gaps persist. While epidemiological studies confirm associations between air pollution and cardiopulmonary outcomes (37, 38), the mechanistic understanding remains limited. Oxidative stress is a key pathway linking pollutant exposure to cardiovascular events (39, 40), but the specific components and mechanisms remain underexplored (41). Limitations include the underrepresentation of vulnerable populations, inadequate exposure characterization, and insufficient data on health effects at low pollutant concentrations (42–44). Future research should focus on identifying more precise biomarkers, enhancing personal exposure assessment, and clarifying how particulate composition influences acute biochemical responses in emergency settings.

Previous reviews have summarized the short-term health effects of air pollution (6), the broad range of biomarkers used in human air-pollution research (34), non-invasive measurement approaches (31, 36), pregnancy-related biomarker studies (33), and metabolomic applications (35). However, these reviews were not designed to map the evidence specifically at the intersection of short-term outdoor exposure, biochemical responses, and acute cardiorespiratory presentations in emergency settings. They also did not systematically compare the exposure windows and lag structures used across this clinically relevant literature. A focused scoping review is therefore needed to clarify what has been studied, how it has been measured, and where the principal methodological gaps remain.

This scoping review aims to systematically map the existing literature on short-term biochemical changes associated with exposure to outdoor air pollution in individuals presenting to emergency departments with acute respiratory or cardiovascular conditions. Specifically, it seeks to identify the types of pollutants and biochemical markers studied, characterize the temporal dynamics of these changes, explore methodological approaches used, and highlight gaps in current knowledge to inform future research and clinical practice.

## 2 Materials and methods

This scoping review was conducted following the methodological framework initially proposed by Arksey and O'Malley (45), and subsequently enhanced by Levac et al. (46) and the Joanna Briggs Institute's updated guidance (47). The review process was designed to ensure comprehensive identification, selection, and synthesis of relevant literature. The reporting of findings adheres to the Preferred Reporting Items for Systematic Reviews and Meta-Analyses extension for Scoping Reviews (PRISMA-ScR) checklist (48) (Supplementary File 1).

### 2.1 Objectives and research questions

The primary objective of this scoping review is to systematically map the existing literature on short-term biochemical changes associated with exposure to outdoor air pollutants in individuals presenting to emergency departments with acute cardiorespiratory conditions. This includes the identification of relevant pollutants and biomarkers, the temporal dynamics of biomarker responses, and the methodological approaches used to assess exposure and biological effects.

Specifically, this review aims to:

- Identify the types of air pollutants and biochemical markers that have been studied in the context of acute cardiovascular or respiratory emergencies.
- Characterize the temporal patterns between pollutant exposure and biochemical alterations preceding or coinciding with emergency presentations.
- Examine the methodologies employed for exposure assessment and biomarker detection in the included studies.
- Explore differences in biomarker responses across disease types (e.g., asthma vs. COPD) and population subgroups (e.g., age, sex, comorbidities).
- Highlight gaps in the current evidence base to inform future research and clinical practice in emergency medicine and environmental health.

Based on these aims, the review is guided by the following research questions:

- RQ1: What biochemical markers have been used to assess short-term physiological responses to air pollution in acute cardiorespiratory emergencies?
- RQ2: Which air pollutants are most commonly associated with these biomarker changes in emergency settings?
- RQ3: What are the typical time intervals between pollutant exposure and measurable biochemical effects?
- RQ4: Which exposure assessment and biomarker measurement methods have been employed?
- RQ5: How do biomarker responses vary across clinical conditions (e.g., asthma vs. myocardial infarction) and demographic subgroups (e.g., children, older adult, individuals with comorbidities)?
- RQ6: What are the major knowledge gaps and methodological limitations identified in the existing literature?

### 2.2 Eligibility criteria

To ensure methodological rigor and transparency, inclusion and exclusion criteria were developed using the PCC framework (Population–Concept–Context), as recommended by the Joanna Briggs Institute for conducting scoping reviews (47, 49). This structured approach allowed for the systematic identification of relevant literature across disciplines including environmental health, emergency medicine, public health, occupational and respiratory medicine.

### 2.3 Search strategy

A comprehensive and systematic search strategy was developed to identify studies across environmental health, emergency medicine, cardiology, pulmonology, and public health. The search concepts were derived from the Population-Concept-Context framework and refined iteratively by combining controlled vocabulary with free-text synonyms for outdoor air pollution, acute cardiorespiratory presentations, emergency-care settings, and biochemical markers. The draft strategy was pilot-tested against known relevant articles before being adapted to the indexing structure and syntax of each database.

Electronic searches were conducted in PubMed, Scopus, Web of Science, Embase, and CINAHL. The search covered publications from January 2000 to September 2025, reflecting the period of increasing attention to acute pollution-related outcomes and biomarker-based assessments.

To maximize retrieval, gray-literature sources, including conference materials and theses or dissertations, were also screened for potentially relevant records and to identify related peer-reviewed publications. In accordance with the pre-defined eligibility criteria, only peer-reviewed original articles meeting all inclusion criteria were retained in the final synthesis.

The database-specific strategies used Boolean operators, truncation symbols, and controlled vocabulary where available. Reference lists of included papers and relevant reviews were checked for additional eligible studies. The complete search strings for each database are provided in Supplementary File 2.

### 2.4 Study selection

Following completion of the search, all references were imported into Rayyan^1^ for screening and deduplication.

The study selection process followed two sequential phases.

#### 2.4.1 Title and abstract screening

Two reviewers (C.L. and D.C.) independently screened all retrieved titles and abstracts based on the eligibility criteria defined in the PCC framework (see Table 1). Studies clearly irrelevant to the research questions were excluded at this stage.

**Table 1:** Eligibility criteria based on the PCC framework.

Component	Criteria	Details
Population	Included	Individuals (adults or children) presenting to emergency departments with acute cardiovascular or respiratory conditions (e.g., asthma exacerbation, COPD flare-up, myocardial infarction, arrhythmia, heart failure)
	Excluded	Studies involving chronic conditions without acute presentation, non-human subjects (e.g., animal or *in vitro* studies), or populations without clear emergency department admission
Concept	Included	Studies reporting biochemical markers (targeted or untargeted) indicative of short-term physiological responses to outdoor air pollution exposure (e.g., inflammation, oxidative stress, myocardial injury markers)
	Excluded	Studies that do not include any biochemical outcome, focus only on clinical or symptomatic endpoints, or assess only long-term effects of pollution
Context	Included	Exposure to ambient (outdoor) air pollution, including urban, peri-urban, and rural environments. Studies using real-world monitoring or modeling approaches
	Excluded	Studies focused exclusively on indoor air pollution or occupational exposures not related to emergency presentations; studies without clear exposure assessment
Study type	Included	Original research articles (e.g., observational studies, case-control, cohort, case-crossover, time-series, clinical studies)
	Excluded	Narrative or systematic reviews, meta-analyses, conference abstracts, editorials, commentaries, theses, and non-peer-reviewed articles
Language	Included	Articles published in English
	Excluded	Studies published in languages other than English

#### 2.4.2 Full-text review

Articles deemed potentially eligible underwent full-text evaluation by both reviewers (C.L. and D.C.). Discrepancies were resolved through discussion or adjudication by a third reviewer when necessary (G.L.).

A PRISMA-ScR flow diagram was used to document the screening and selection process, including the number of records identified, screened, excluded, and included. Reasons for exclusion during full-text review were systematically recorded to ensure methodological transparency.

The final selection included studies that directly examined the association between outdoor air pollution and acute biochemical responses, specifically in patients presenting with emergency cardiovascular or respiratory conditions.

### 2.5 Data charting and synthesis

Data were charted independently by two reviewers (C.L. and D.C.) using a standardized extraction form that was piloted on a subset of eligible studies and refined by consensus. Any disagreement was resolved by discussion, with adjudication by a third reviewer (G.L.) when required. Extracted items included author and year, study design, population and sample size, acute clinical context, pollutants and exposure-assessment methods, exposure windows and lag structures, biological specimen and biomarker-assay methods when reported, biomarkers evaluated, direction and magnitude of associations, measures of precision or statistical significance, subgroup analyses, and study-level limitations.

Consistent with the objectives of a scoping review, the evidence was mapped descriptively rather than pooled meta-analytically. Studies were grouped by clinical population, pollutant, biomarker domain, and reported exposure window. Biomarker domains included inflammation, coagulation or endothelial function, cardiac injury or stress, immune or hematological response, and oxidative stress or antioxidant capacity. Counts and percentages were calculated using the 12 included studies as the denominator; categories were not mutually exclusive. Exposure windows were summarized as intraday or same-day, lagged 1–3 days, multiday or cumulative, or not clearly reported. No formal risk-of-bias appraisal was undertaken because the purpose was to map the extent, characteristics, and gaps of the evidence base.

## 3 Results

**Figure 1:**
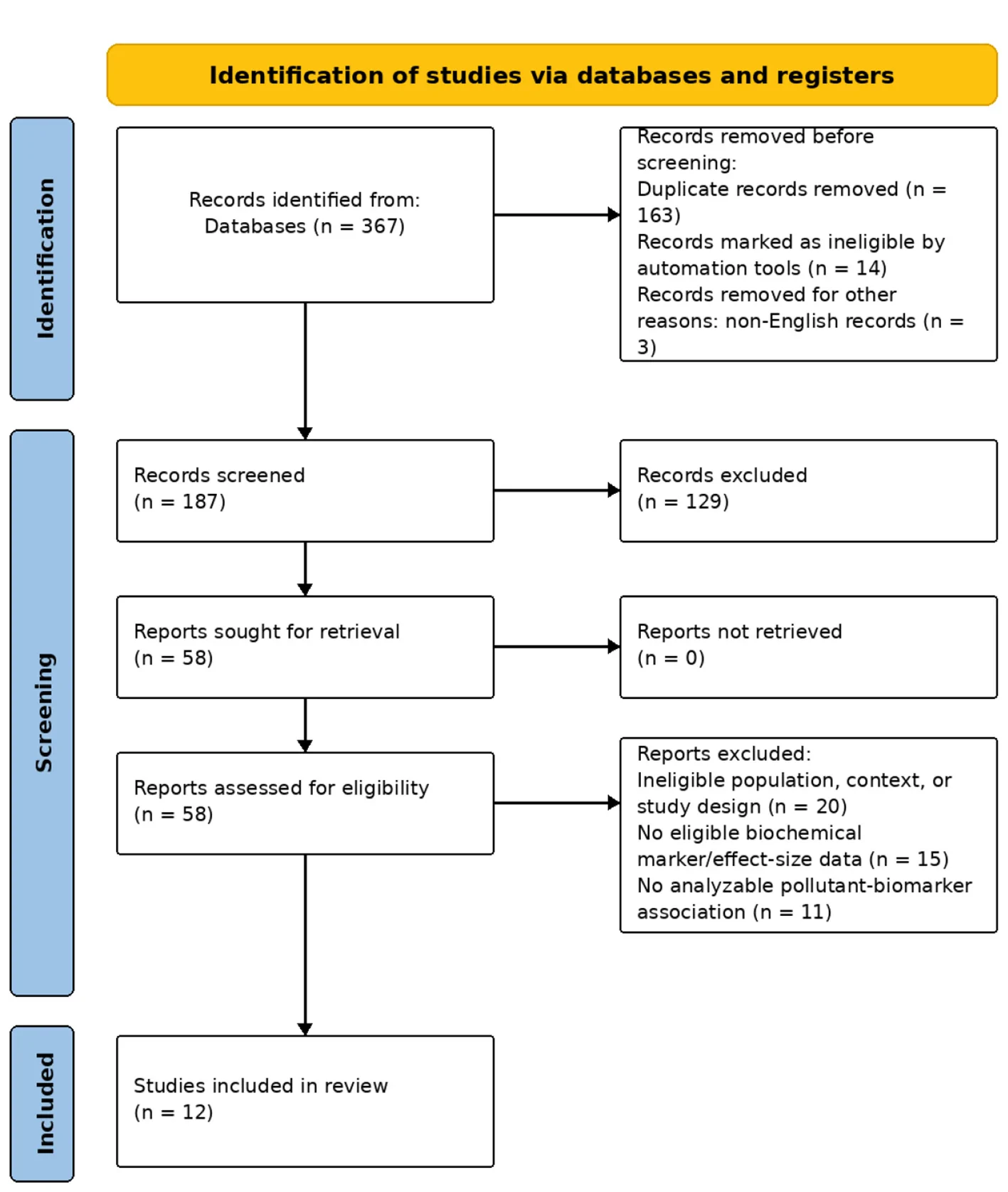
PRISMA-ScR flow diagram illustrating the identification, screening, eligibility, and inclusion of studies.

A comprehensive search across the electronic databases yielded 367 records. As shown in Figure 1, 180 records were removed before screening: 163 duplicates, 14 records marked as ineligible by automation tools, and three records removed for other reasons (non-English records). The remaining 187 records underwent title and abstract screening, and 129 were excluded. Fifty-eight full-text reports were assessed for eligibility. Of these, 46 were excluded: 20 because the population, context, or study design was not eligible; 15 because eligible biochemical marker or effect-size data were not available; and 11 because no analyzable pollutant-biomarker association was reported. Twelve studies were included in the final descriptive synthesis.

Figure 1 presents the PRISMA-ScR flow diagram, summarizing the identification, screening, eligibility assessment, and inclusion of studies, together with the main reasons for exclusion at the full-text stage.

The included studies span various methodological designs—cross-sectional, cohort, case-control, and longitudinal—and encompass diverse patient populations, ranging from myocardial infarction and COPD to COVID-19 and acute coronary syndromes. Air pollutants most frequently examined included particulate matter (PM_2.5_, PM10), nitrogen oxides (NO, NO_2_), ozone (O_3_), sulfur dioxide (SO_2_), and benzo[a]pyrene (B[a]P).

The biochemical markers assessed covered a wide range of inflammatory, coagulatory, and myocardial injury-related biomarkers, such as CRP, IL-6, D-dimer, fibrinogen, procalcitonin, and cardiac troponins. Across studies, associations between pollutant exposure and biomarker levels were frequently found to be statistically significant, with effect sizes reported as odds ratios, mean differences, and correlation coefficients.

### 3.1 Characteristics of included studies

The 12 included studies used heterogeneous observational designs, including cross-sectional, cohort, case-crossover, case-control, and longitudinal approaches. Sample sizes ranged from 38 people with chronic pulmonary disease (50) to more than 210,000 emergency-room admissions (51). The clinical settings also differed substantially, spanning acute coronary syndromes, myocardial infarction or angina, chronic pulmonary disease, acute exacerbations of COPD, COVID-19 hospitalization, and respiratory emergency-room visits. This heterogeneity broadens the scope of the map but limits direct comparison of effect estimates.

Table 2 summarizes the main characteristics of the included studies, including study design, population and sample size, clinical context, pollutants examined, biomarkers evaluated, and reported associations.

**Table 2:** Summary of included studies: pollutants, biomarkers, and acute cardiorespiratory outcomes.

Author and year	Study design	Study population	Atmospheric pollutants examined	Biomarkers evaluated	Effect size	Statistical significance
Bedada (57)	Case-crossover; longitudinal	709 (transient ischemic attack), 35 (diabetes), 28,622 (acute coronary syndrome)	PM_10_, SO_2_, NO, NO_2_, PM_2.5_ (personal)	cardiac troponin T (cTnT), interleukin-6 (IL-6), high-sensitivity C-reactive protein (hs-CRP), fibrinogen	Odds ratio 1.14 (PM10), odds ratio 1.11 (SO2), significant interleukin-6 with personal PM_2.5_	95% confidence interval: 1.05–1.24, 1.00–1.23; interleukin-6 significant
Camacho et al. (51)	Retrospective; cross-sectional	210,540 emergency room admissions; older adults	Nitrogen oxides (NO_x_), O_3_, PM_2.5_, SO_2_	Creatine kinase MB, B-type natriuretic peptides	No effect size	Significant association
Cecconi et al. (58)	Cross-sectional	249 people with myocardial infarction or angina; mean age 62–67; mostly male	Particulate matter less than 10 micrometers (PM_10_), particulate matter less than 2.5 micrometers (PM_2.5_), nitrogen dioxide (NO_2_), sulfur dioxide (SO_2_), nitric oxide (NO), carbon monoxide (CO), ozone (O_3_)	CD4, CD25, Foxp3, interleukin-22 (IL-22), interferon gamma (IFNγ), interleukin-17A (IL-17A), microRNAs, troponin T, creatine kinase	Correlation coefficient = −0.18 (CD4+ CD69+ vs. PM2.5), 0.27 (CO vs. CD4+), −0.19 (SO2 vs. leukocytes)	Significant association
Croft et al. (52)	Cross-sectional	135 people undergoing cardiac catheterization	Delta-C, black carbon, ultrafine particles, accumulation mode particles, PM_2.5_	CRP, D-dimer, fibrinogen, P-selectin, platelet factor 4 (PF-4), von Willebrand factor (vWF), myeloperoxidase (MPO)	Fibrinogen: 0.91% increase per 0.13 micrograms/m^3^ Delta-C; myeloperoxidase: 2.75% decrease per 0.17 micrograms/m^3^ Delta-C	95% confidence interval: 0.23–1.59% (fibrinogen), −5.13 to −0.37% (myeloperoxidase)
Díaz-Chirón et al. (59)	Prospective cohort	216 people with acute coronary syndrome; mean age 62–66; ~81% male	SO_2_, NO, NO_2_, CO, O_3_, benzene, toluene, xylene, particulate matter (PM)	Hemoglobin, leukocytes, neutrophils, lymphocytes, platelets, troponin T, neutrophil-to-lymphocyte ratio (NLR)	Odds ratio 1.12 (SO2), odds ratio 1.08 (NLR)	*p* = 0.007, 0.024; 95% confidence interval: 1.031–1.21, 1.011–1.17
Hildebrandt et al. (50)	Longitudinal	38 men with chronic pulmonary disease; mean age 54	Ultrafine particles, accumulation mode particles, PM10, elemental carbon, organic carbon, NO, NO_2_, CO, SO_2_	Fibrinogen, CRP, serum amyloid A (SAA), E-selectin, soluble intercellular adhesion molecule-1 (sICAM-1), vWF, prothrombin fragment 12 + (F12 + ), D-dimer, factor VII (FVII)	Fibrinogen increased (lag 3), E-selectin increased (lag 1), von Willebrand factor decreased (lags 1–4), prothrombin fragment 12 + decreased (lag 4)	Significant
Lee et al. (60)	Cohort; longitudinal	6,589 non-smokers	PM_2.5_, PM_10_, NO_2_, SO_2_, O_3_, CO	Fibrinogen, CRP, ferritin, white blood cell count (WBC)	Ferritin: 1.4% increase (NO2); fibrinogen: 3.4% increase (PM2.5)	95% confidence interval: 0.3–2.5% (ferritin), 3.0–3.8% (fibrinogen)
Panasevich et al. (61)	Case-control; nested case-crossover	2,698 (myocardial infarction cases/controls); 45–70 years	Traffic-related NO_2_, heating-related SO_2_, NO_2_, SO_2_, PM_10_, O_3_	IL-6, tumor necrosis factor alpha (TNF-α), fibrinogen, plasminogen activator inhibitor-1 (PAI-1)	Genotype-specific increases in interleukin-6, tumor necrosis factor alpha	Significant
Poniedziałek et al. (53)	Cross-sectional	2,225 people hospitalized with COVID-19	PM_10_, PM_2.5_, benzo(a)pyrene (B(a)P)	CRP, IL-6, D-dimer, procalcitonin	CRP: OR = 1.78 (B[a]P); IL-6: OR = 1.71 (PM_2.5_); D-dimer: OR = 1.62 (B[a]P); Procalcitonin: OR = 1.41 (PM_10_)	CRP, IL-6, D-dimer: *p* < 0.001; Procalcitonin: *p* < 0.01
Rückerl et al. (55)	Longitudinal	1,003 people who survived myocardial infarction; 35–80 years	Particle number concentration (PNC), PM_10_, PM_2.5_, gases	IL-6, fibrinogen, CRP	Interleukin-6: 2.7% increase (particle number concentration); fibrinogen: 0.6% increase (PM_10_)	95% confidence interval: 1.0–4.6% (interleukin-6), 0.1–1.1% (fibrinogen)
Tang et al. (54)	Cohort	317 people hospitalized with acute exacerbation of chronic obstructive pulmonary disease; 71%≥65; 80% male	PM_2.5_, PM_10_	C-reactive protein (CRP), D-dimer, thrombin time (TT), prothrombin time (PT), fibrinogen (FIB), activated partial thromboplastin time (APTT)	C-reactive protein: mean difference 3.03 mg/L (PM_2.5_), 3.75 mg/L (PM_10_); prothrombin time: 12.03 ± 1.89 s	*p* = 0.046, 0.009, 0.041; odds ratio 2.19 (C-reactive protein ≥10 mg/L, PM_2.5_ ≥25 micrograms/m^3^)
Weichenthal et al. (62)	Case-crossover	No mention found (large emergency room dataset)	PM_2.5_ (oxidative potential)	Glutathione, ascorbate (synthetic fluid)	Effect modification by glutathione-related oxidative potential	*p* = 0.001 (at low PM_2.5_)

APTT, activated partial thromboplastin time; B(a)P/B[a]P, benzo[a]pyrene; BNP, B-type natriuretic peptide; CD4, cluster of differentiation 4; CD25, cluster of differentiation 25; CD69, cluster of differentiation 69; CK-MB, creatine kinase-MB; CO, carbon monoxide; CRP, C-reactive protein; cTnT, cardiac troponin T; FIB, fibrinogen; F12+, prothrombin fragment 1+2; FVII, factor VII; Foxp3, forkhead box P3; hs-CRP, high-sensitivity C-reactive protein; IFN-γ, interferon gamma; IL-6, interleukin-6; IL-17A, interleukin-17A; IL-22, interleukin-22; MPO, myeloperoxidase; NLR, neutrophil-to-lymphocyte ratio; NO, nitric oxide; NO_2_, nitrogen dioxide; NOx, nitrogen oxides; O_3_, ozone; OR, odds ratio; PAI-1, plasminogen activator inhibitor-1; PF-4, platelet factor 4; PM, particulate matter; PM_2.5_, particulate matter with an aerodynamic diameter ≤ 2.5 μm; PM_10_, particulate matter with an aerodynamic diameter ≤ 10 μm; PNC, particle number concentration; PT, prothrombin time; SAA, serum amyloid A; sICAM-1, soluble intercellular adhesion molecule-1; SO_2_, sulfur dioxide; TNF-α, tumor necrosis factor alpha; TT, thrombin time; vWF, von Willebrand factor; WBC, white blood cell count; Delta-C, difference in light absorption between selected wavelengths.

PM_2.5_ and PM10 were the dominant exposures, while NO_2_, SO_2_, O_3_, CO, ultrafine particles, particle number concentration, Delta-C, and benzo[a]pyrene were evaluated less consistently. The most frequently assessed biochemical pathways were inflammation and coagulation or endothelial dysfunction. Cardiac injury or stress markers, immune-cell profiles, and oxidative-potential or antioxidant metrics were evaluated in smaller subsets of studies.

Table 3 provides a study-level evidence map that integrates sample size, clinical population or context, key age and subgroup details when reported, pollutants assessed, and biomarker domains.

**Table 3:** Study-level descriptive evidence map of included studies (*N* = 12), including sample size and key population characteristics when reported. NR, not reported or not clearly extractable from the source article.

Study	Sample size	Clinical population/context	Age/sex or key subgroup details	Pollutant or exposure metric	Biomarker domain(s)
Bedada (57)	709 transient ischemic attack; 35 diabetes; 28,622 acute coronary syndrome	Transient ischemic attack, diabetes subgroup, and acute coronary syndrome	NR in extracted data	PM10, SO_2_, NO, NO_2_, personal PM_2.5_	Cardiac injury; inflammation; coagulation
Camacho et al. (51)	210,540 emergency-room admissions	Emergency-room admissions for acute cardiorespiratory outcomes	Adults and older adults; further subgroup details not clearly reported	NOx, O_3_, PM_2.5_, SO_2_	Cardiac injury or stress
Cecconi et al. (58)	249	People with myocardial infarction or angina	Mean age 62–67 years; mostly male	PM10, PM_2.5_, NO_2_, SO_2_, NO, CO, O_3_	Immune or hematological; cardiac injury
Croft et al. (52)	135	People undergoing cardiac catheterization	NR in extracted data	Delta-C, black carbon, ultrafine particles, accumulation-mode particles, PM_2.5_	Inflammation; coagulation or endothelial function
Díaz-Chirón et al. (59)	216	People with acute coronary syndrome	Mean age 62–66 years; approximately 81% male	SO_2_, NO, NO_2_, CO, O_3_, volatile organic pollutants, PM	Immune or hematological; cardiac injury
Hildebrandt et al. (50)	38	Men with chronic pulmonary disease	Mean age 54 years; 100% male	Ultrafine particles, accumulation-mode particles, PM10, carbon fractions, gaseous pollutants	Inflammation; coagulation or endothelial function
Lee et al. (60)	6,589	Non-smokers	NR in extracted data	PM_2.5_, PM10, NO_2_, SO_2_, O_3_, CO	Inflammation; immune or hematological
Panasevich et al. (61)	2,698	Myocardial infarction cases and controls	Age range 45–70 years	Traffic-related NO_2_; heating-related SO_2_; PM10; O_3_	Inflammation; coagulation
Poniedziałek et al. (53)	2,225	People hospitalized with COVID-19	NR in extracted data	PM10, PM_2.5_, benzo[a]pyrene	Inflammation; coagulation
Rückerl et al. (55)	1,003	People who survived myocardial infarction	Age range 35–80 years	Particle-number concentration, PM10, PM_2.5_, gases	Inflammation; coagulation
Tang et al. (54)	317	People hospitalized with acute exacerbation of COPD	71% aged ≥65 years; 80% male	PM_2.5_, PM10	Inflammation; coagulation
Weichenthal et al. (62)	Large ER dataset; exact n NR in extracted data	Respiratory emergency-room visits	NR in extracted data	PM_2.5_ oxidative potential	Oxidative-potential or antioxidant metrics

CO, carbon monoxide; COPD, chronic obstructive pulmonary disease; COVID-19, coronavirus disease 2019; ER, emergency room; NO, nitric oxide; NO_2_, nitrogen dioxide; NOx, nitrogen oxides; NR, not reported or not clearly extractable from the source article; O_3_, ozone; PM, particulate matter; PM_2.5_, particulate matter with an aerodynamic diameter ≤ 2.5 μm; PM_10_, particulate matter with an aerodynamic diameter ≤ 10 μm; SO_2_, sulfur dioxide; Delta-C, difference in light absorption between selected wavelengths.

Effect estimates were reported in several formats, including odds ratios, mean differences, correlation coefficients, and percentage changes. For example, fibrinogen and myeloperoxidase changed in relation to Delta-C exposure (52), inflammatory and coagulation markers were associated with short-term PM and benzo[a]pyrene exposure in people hospitalized with COVID-19 (53), and CRP and coagulation parameters changed in people hospitalized with acute exacerbations of COPD (54). These findings should be interpreted as a map of reported associations rather than as pooled evidence of a uniform effect.

**Figure 2:**
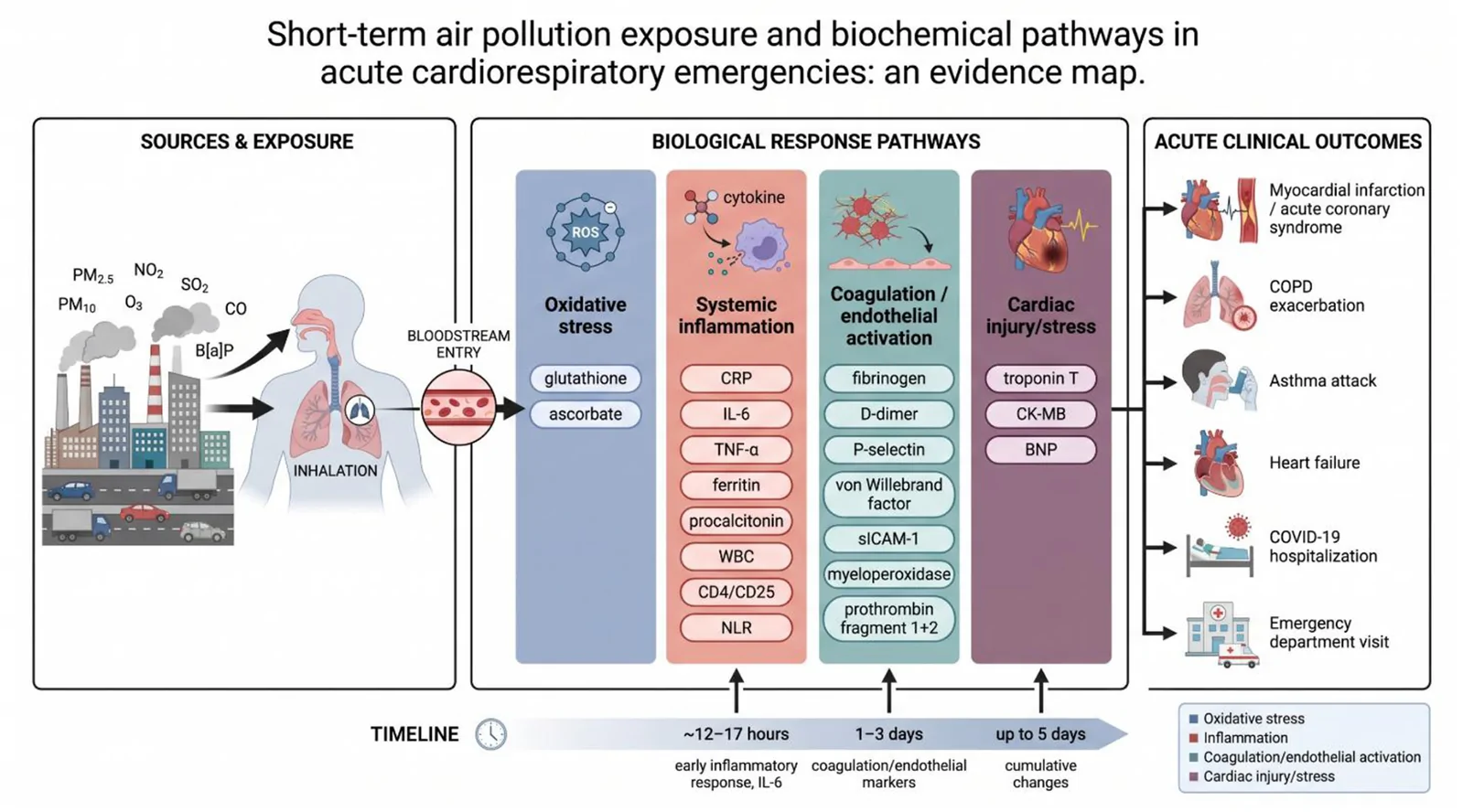
Short-term air pollution exposure and biochemical pathways in acute cardiorespiratory emergencies: an evidence map. Created in BioRender. Ledda, C. (2026) https://BioRender.com/19mpigo.

Temporal reporting was uneven. Some studies specified lagged responses, including early changes in IL-6 after particle-number exposure and later changes in fibrinogen, as well as lagged changes in E-selectin, von Willebrand factor, and prothrombin fragment 12 + (50, 55). In other reports, the exposure window was described only broadly as short-term. Table 4 separates explicitly reported lag structures from windows that were not clearly specified, while Figure 2 provides a visual synthesis of the pollutants, biological pathways, biomarkers, and temporal windows identified across the included studies, situating these lag structures within the broader exposure–response framework.

**Table 4:** Exposure windows and lagged biochemical associations reported in the included studies.

Study	Pollutant or exposure metric	Reported exposure window or lag	Biomarker(s)	Main reported biochemical association
Bedada (57)	PM10, SO_2_, NO, NO_2_, personal PM_2.5_	Short-term exposure; exact lag not clearly reported	cTnT, IL-6, hs-CRP, fibrinogen	PM10 and SO_2_ associated with increased cTnT; personal PM_2.5_ associated with IL-6.
Camacho et al. (51)	NOx, O_3_, PM_2.5_, SO_2_	Exact window not clearly reported	CK-MB, BNP	Significant associations reported for cardiac biomarkers; effect sizes not reported.
Cecconi et al. (58)	PM10, PM_2.5_, NO_2_, SO_2_, NO, CO, O_3_	Short-term exposure; exact lag not clearly reported	Immune-cell markers, cytokines, microRNAs, troponin T, creatine kinase	Mixed immunomodulatory correlations, including inverse associations for PM_2.5_ and SO_2_ and a positive association for CO.
Croft et al. (52)	Delta-C, black carbon, ultrafine particles, accumulation-mode particles, PM_2.5_	Short-term exposure; exact lag not clearly reported	CRP, D-dimer, fibrinogen, P-selectin, PF-4, vWF, MPO	Fibrinogen increased by 0.91% and MPO decreased by 2.75% per specified Delta-C increments.
Díaz-Chirón et al. (59)	SO_2_, NO, NO_2_, CO, O_3_, volatile organic pollutants, PM	Short-term exposure; exact lag not clearly reported	Blood-cell markers, troponin T, NLR	SO_2_ and NLR were associated with the reported acute-coronary outcomes.
Hildebrandt et al. (50)	Ultrafine particles, accumulation-mode particles, PM10, carbon fractions, gaseous pollutants	Lag 1–4 days	Fibrinogen, CRP, SAA, E-selectin, sICAM-1, vWF, F12 +, D-dimer, FVII	Fibrinogen increased at lag 3; E-selectin increased at lag 1; vWF decreased at lags 1–4; F12 + decreased at lag 4.
Lee et al. (60)	PM_2.5_, PM10, NO_2_, SO_2_, O_3_, CO	Short- and long-term windows; exact lag not clearly reported in this review	Fibrinogen, CRP, ferritin, WBC	Ferritin increased with NO_2_ and fibrinogen increased with PM_2.5_.
Panasevich et al. (61)	Traffic-related NO_2_; heating-related SO_2_; PM10; O_3_	Short-term exposure; exact lag not clearly reported	IL-6, TNF-α, fibrinogen, PAI-1	Genotype-specific changes in IL-6 and TNF-α.
Poniedziałek et al. (53)	PM10, PM_2.5_, benzo[a]pyrene	Short-term exposure; exact lag not clearly reported	CRP, IL-6, D-dimer, procalcitonin	Higher CRP, IL-6, D-dimer, and procalcitonin were associated with pollutant exposure.
Rückerl et al. (55)	Particle-number concentration, PM10, PM_2.5_, gases	Approximately 12–17 h for IL-6; multiday cumulative window for fibrinogen	IL-6, fibrinogen, CRP	IL-6 increased by 2.7% with particle-number concentration; fibrinogen increased by 0.6% with PM10.
Tang et al. (54)	PM_2.5_, PM10	Short-term exposure; exact lag not clearly reported	CRP, D-dimer, TT, PT, fibrinogen, APTT	CRP increased and coagulation parameters changed during acute COPD exacerbations.
Weichenthal et al. (62)	PM_2.5_ oxidative potential	Short-term exposure; exact lag not clearly reported	Glutathione- and ascorbate-related oxidative-potential metrics	Oxidative potential modified the association between PM_2.5_ and respiratory emergency-room visits.

NR-style wording (“not clearly reported”) indicates that a directly comparable exposure window or lag could not be extracted from the source summary available in the manuscript. APTT, activated partial thromboplastin time; BNP, B-type natriuretic peptide; CK-MB, creatine kinase-MB; CO, carbon monoxide; COPD, chronic obstructive pulmonary disease; CRP, C-reactive protein; cTnT, cardiac troponin T; F12+, prothrombin fragment 1+2; FVII, factor VII; hs-CRP, high-sensitivity C-reactive protein; IL-6, interleukin-6; MPO, myeloperoxidase; NLR, neutrophil-to-lymphocyte ratio; NO, nitric oxide; NO_2_, nitrogen dioxide; NOx, nitrogen oxides; NR, not reported; O_3_, ozone; PAI-1, plasminogen activator inhibitor-1; PF-4, platelet factor 4; PM, particulate matter; PM_2.5_, particulate matter with an aerodynamic diameter ≤ 2.5 μm; PM_10_, particulate matter with an aerodynamic diameter ≤ 10 μm; PT, prothrombin time; SAA, serum amyloid A; sICAM-1, soluble intercellular adhesion molecule-1; SO_2_, sulfur dioxide; TNF-α, tumor necrosis factor alpha; TT, thrombin time; vWF, von Willebrand factor; WBC, white blood cell count; Delta-C, difference in light absorption between selected wavelengths.

### 3.2 Synthesis of research questions

To address the objectives of this scoping review, the findings from the included studies were synthesized according to six guiding research questions (RQ1–RQ6).

- RQ1: What biochemical markers have been used to assess short-term physiological responses to air pollution in acute cardiorespiratory emergencies?
  The most frequently used biomarkers across the included studies were inflammatory markers such as C-reactive protein (CRP), interleukin-6 (IL-6), and fibrinogen, along with coagulatory indicators like D-dimer and prothrombin fragments. Cardiac injury biomarkers were also commonly evaluated, notably troponin T, CK-MB, and B-type natriuretic peptide (BNP). Some studies explored oxidative stress (e.g., glutathione, ascorbate) and immune modulation (e.g., CD4/CD25 expression), while others assessed composite hematological markers such as neutrophil-to-lymphocyte ratio (NLR) and platelet counts. Together, these biomarkers reflect systemic inflammation, coagulation dysregulation, and myocardial injury.
- RQ2: Which air pollutants are most commonly associated with these biomarker changes in emergency settings?
  Particulate matter (PM_2.5_ and PM10) was by far the most frequently reported pollutant across studies, showing consistent associations with CRP, IL-6, fibrinogen, and cardiac enzymes. Nitrogen dioxide (NO_2_) was also commonly studied, often linked with increases in inflammatory markers such as IL-6 and ferritin. Additional pollutants included ozone (O_3_), sulfur dioxide (SO_2_), carbon monoxide (CO), benzo[a]pyrene (B[a]P), and ultrafine particles, with varied but often significant biochemical impacts.
- RQ3: What are the typical time intervals between pollutant exposure and measurable biochemical effects?
  The reported exposure windows ranged from hours to several days, but they were not standardized across studies. Early inflammatory changes were described within approximately 12–17 h after elevated particle-number concentrations, whereas fibrinogen and endothelial or coagulation-related markers were reported across 1- to 5-day windows in selected studies. Several investigations referred to short-term exposure without reporting a directly comparable lag definition. This heterogeneity is summarized in Table 4 and should be considered when comparing results across pollutants and clinical populations.
- RQ4: Which exposure assessment and biomarker measurement methods have been employed?
  Exposure assessment methods pre-dominantly relied on data from ambient air quality monitoring stations, providing regional estimates of pollutant concentrations. In some cases, these were complemented by individualized exposure models to improve spatial accuracy. A limited number of studies employed more advanced indicators, such as pollutant oxidative potential or 24-h mean concentrations, to better capture short-term fluctuations in air quality. Biomarkers were typically measured from venous blood samples collected in emergency or inpatient settings. Standard laboratory assays were used to quantify key inflammatory and cardiac markers, including C-reactive protein, interleukin-6, troponins, and D-dimer. In studies focusing on immune modulation, more specialized techniques, such as flow cytometry or enzyme-linked immunosorbent assays (ELISA), were applied to evaluate specific cell surface markers and cytokine levels.
- RQ5: How do biomarker responses vary across clinical conditions and demographic subgroups?
  Biomarker responses varied considerably depending on the clinical condition of the patients. Individuals with chronic obstructive pulmonary disease typically exhibited elevated levels of C-reactive protein and prolonged coagulation times, indicating a heightened inflammatory and thrombotic state. In people hospitalized for COVID-19, exposure to particulate matter and polycyclic aromatic hydrocarbons such as benzo[a]pyrene was associated with pronounced increases in inflammatory and coagulation biomarkers, reflecting a systemic response to environmental stressors. Those presenting with myocardial infarction or angina showed distinct immunological changes, including alterations in T cell populations and elevated fibrinogen or interleukin-6 concentrations. Despite the inclusion of older adults and individuals with pre-existing conditions in several studies, few analyses stratified results by demographic or clinical subgroups, highlighting a persistent gap in understanding how vulnerability factors influence biochemical responses to air pollution.
- RQ6: What are the major knowledge gaps and methodological limitations identified in the existing literature?
  Several limitations emerged across the studies. First, inconsistent lag-time reporting limits comparability across datasets. Second, exposure assessments were largely ecological, lacking personal or indoor exposure validation. Third, few studies included vulnerable populations such as children, pregnant women, or racial/ethnic minorities. Also, longitudinal biochemical tracking was rare, and baseline levels were often not reported. Lastly, although over a dozen biomarkers were assessed, integrative biomarker panels or multi-omics approaches remain underutilized. These limitations highlight the need for standardized methods, better temporal resolution, and demographic stratification in future research.

## 4 Discussion

This scoping review maps a clinically relevant but methodologically heterogeneous literature on short-term outdoor air-pollution exposure and biochemical responses in acute cardiorespiratory settings. The evidence base is dominated by particulate matter and by inflammatory and coagulation-related markers. The findings support biological plausibility for rapid systemic responses, but they should not be interpreted as demonstrating a single, uniform exposure-response pattern across all pollutants, biomarkers, or emergency presentations.

The overall pattern is compatible with an oxidative-stress and inflammatory pathway in which pulmonary exposure can contribute to systemic mediator release and downstream vascular effects (39, 40, 56). The lagged findings are potentially informative: IL-6 may reflect an earlier response, while CRP, fibrinogen, endothelial markers, and selected coagulation markers may change over subsequent days. However, this interpretation remains provisional because exposure windows, lag definitions, and biomarker-sampling schedules were not harmonized across studies (63–82).

The clearest limitation of the underlying evidence is comparability. Studies differed in clinical populations, sample size, pollutant mixtures, exposure metrics, analytical methods, and reported effect measures. Most exposure assessments relied on fixed-site ambient monitoring, which can misclassify individual exposure, particularly when indoor environments, mobility, and spatial variability are not captured. More refined models, personal monitoring, and complementary biomonitoring approaches may reduce this limitation (31, 36).

The clinical implications should therefore be framed cautiously. Biomarkers such as CRP, IL-6, fibrinogen, D-dimer, and troponins are biologically informative, but the current evidence does not support their routine use as pollution-specific diagnostic tools in emergency departments. Their nearer-term value lies in research, surveillance, and hypothesis-driven risk stratification, particularly when biomarker sampling is paired with high-resolution exposure data and clearly pre-specified lag windows.

Important evidence gaps remain. Few studies stratified results by age, sex, socioeconomic conditions, or comorbidity burden, despite the potential for differential susceptibility. Children, pregnant people, and other potentially vulnerable populations were underrepresented. Oxidative-stress markers, immune phenotyping, non-invasive biomarkers, and multi-omics approaches were also used infrequently. These gaps are consistent with broader calls for improved characterization of particulate composition, low-concentration effects, and susceptible populations (41–44).

This review has several strengths. It focuses on the intersection of environmental exposure, biochemical response, and acute clinical presentation; applies an explicit PCC framework; reports the selection process using PRISMA-ScR; and maps study characteristics, biomarker domains, and exposure windows in structured tables. The new descriptive evidence map and lag-summary table make the heterogeneity of the literature more transparent and identify where apparently similar findings arise from non-comparable methods.

This review also has limitations. It was restricted to English-language peer-reviewed original articles published from 2000 to September 2025. Gray-literature records were screened to improve retrieval but were not retained in the final synthesis unless a corresponding eligible peer-reviewed article was available. No formal risk-of-bias appraisal or meta-analysis was undertaken, in keeping with the mapping purpose of the scoping review. Finally, some exposure windows and lag structures were incompletely reported in the source studies, limiting the precision of the temporal synthesis.

Future studies should use pre-specified and comparable exposure windows, report lag-specific estimates with measures of precision, incorporate personal or high-resolution exposure assessment, and evaluate harmonized biomarker panels across clinically defined subgroups. Such improvements would help determine whether biochemical markers can support timely prevention strategies and more precise environmental-health surveillance in emergency-care settings.

## 5 Conclusions

This scoping review identifies a heterogeneous but clinically relevant body of evidence linking short-term outdoor air-pollution exposure with biochemical changes in acute cardiorespiratory settings. PM_2.5_ and PM10 were the most frequently examined pollutants, and inflammatory and coagulation-related markers were the most commonly assessed biomarker domains. Reported responses occurred across windows ranging from hours to several days, but lag definitions and exposure-assessment methods varied substantially. The available evidence supports further investigation of biochemical markers as tools for environmental-health research and surveillance rather than their immediate adoption as pollution-specific diagnostic tests in emergency care. Future studies should use harmonized biomarker panels, clearly pre-specified lag structures, high-resolution or personal exposure measures, and subgroup analyses focused on populations with greater susceptibility.

## Footnotes

1 rayyan. (2024). Available online at: https://www.rayyan.ai/

## Supplementary material

Supplementary File 1 Scoping reviews (PRISMA-ScR) checklist.

Supplementary File 2 Complete search strategy for all electronic databases (PubMed, Scopus, Web of Science, Embase, CINAHL), including grey-literature screening terms.
