# Supplementary material for: Tracking biochemical indicators of air pollution exposure in acute cardiorespiratory emergencies: a scoping review
Source: Front Public Health. 2026 Sep 10;14:1892038. doi: 10.3389/fpubh.2026.1892038 (PMC13602121; doi:10.3389/fpubh.2026.1892038)
Supplement: Supplementary file 1 [file Table_1.docx]

Supplementary Table. Search Strategies by Database

| **Database** | **Search Strategy (Boolean Operators + Keywords)** | **Notes** |
| --- | --- | --- |
| **PubMed** | ("air pollution"[MeSH] OR "particulate matter"[MeSH] OR PM2.5 OR NO2 OR ozone OR "fine particles") AND ("biomarkers"[MeSH] OR "biochemical markers" OR "inflammatory markers" OR "oxidative stress" OR troponin OR "C-reactive protein" OR cytokines OR metabolomics) AND ("emergency department" OR "acute illness" OR "hospitalization" OR "asthma exacerbation" OR "myocardial infarction" OR "COPD exacerbation") | Uses MeSH and free-text terms; limited to human studies published from 2000 to 2025 |
| **Scopus** | TITLE-ABS-KEY(("air pollution" OR "particulate matter" OR PM2.5 OR NO2 OR ozone) AND ("biochemical marker" OR biomarker OR troponin OR "oxidative stress" OR "C-reactive protein") AND ("emergency" OR "acute" OR hospitalization OR asthma OR "COPD" OR "heart failure")) | Searches titles, abstracts, and keywords; filters for document type: article |
| **Web of Science** | TS=("air pollution" OR "particulate matter" OR PM2.5 OR NO2 OR ozone) AND TS=("biomarker" OR "inflammatory marker" OR troponin OR "oxidative stress" OR cytokines OR metabolomics) AND TS=("emergency" OR "acute" OR "hospital admission" OR asthma OR COPD OR myocardial infarction) | TS = topic search (title, abstract, keywords); no language restrictions applied |
| **Embase** | ('air pollution'/exp OR 'particulate matter'/exp OR PM2.5 OR NO2 OR ozone) AND ('biomarker'/exp OR 'biochemical marker' OR 'oxidative stress' OR troponin OR 'inflammatory marker') AND ('emergency care'/exp OR 'acute disease'/exp OR asthma OR COPD OR 'myocardial infarction') | Uses EMTREE terms and keywords; filters for human studies only |
| **CINAHL (EBSCO)** | ("air pollution" OR "particulate matter" OR PM2.5 OR NO2 OR ozone) AND ("biomarker" OR "oxidative stress" OR "inflammatory marker" OR troponin OR cytokines) AND ("emergency department" OR acute OR asthma OR "hospital admission" OR "cardiac event") | Searches titles and subject headings; limited to peer-reviewed human studies |
| **Google Scholar** | allintitle: air pollution biomarker emergency OR “air pollution” AND “biochemical marker” AND “emergency department” | Used to identify grey literature and academic reports; first 200 results screened |
| **OpenGrey** | "air pollution" AND biomarker AND emergency | Grey literature source; relevant reports screened for inclusion |
